# Supplementary material for: Surface Area of Wood Influences the Effects of Fungal Interspecific Interaction on Wood Decomposition—A Case Study Based on Pinus densiflora and Selected White Rot Fungi
Source: J Fungi (Basel). 2022 May 18;8(5):517. doi: 10.3390/jof8050517 (PMC9145481; doi:10.3390/jof8050517)
Supplement: Supplementary file 1 [file jof-08-00517-s001.zip › jof-1721352-supplementary.pdf]

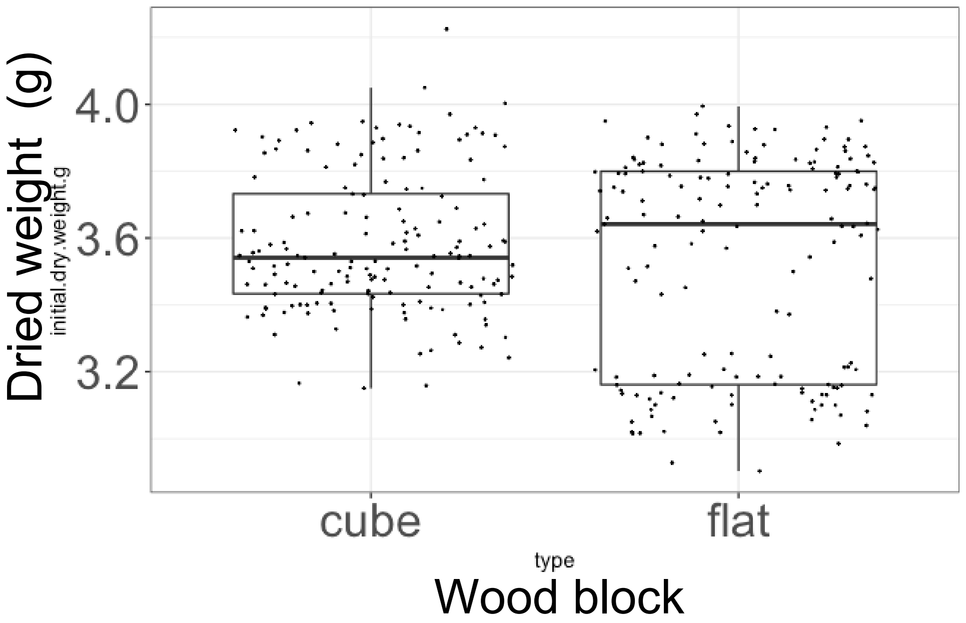

Figure S1 Dried weight of the cube and flat wood blocks before fungal inoculation. Significant difference between cube and flat was not detected by Wilcoxon rank-sum test ($P < 0.05$). N = 159.

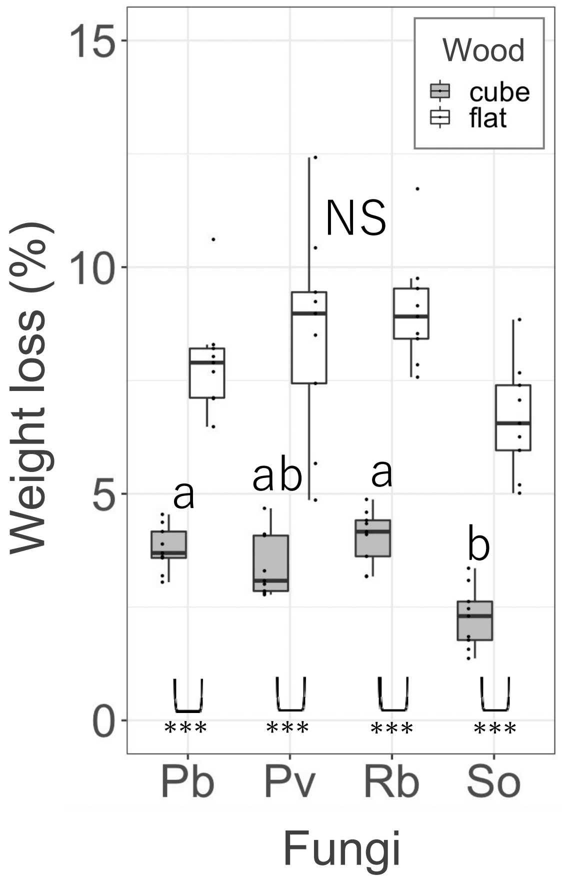

Figure S2 Weight loss of wood blocks after 3 months incubation in pure culture of the four fungal strains. NS and the same alphabets indicate no significant differences among fungal strains in the post hoc Nemenyi test (uppercase, flat; lowercase, cube). Comparison between flat and cube blocks within a strain was performed using the Wilcoxon rank sum test (***, $P < 0.001$). N = 6.
